# Open Auditorium—A human-centric extended reality blended learning tool

**DOI:** 10.3389/frai.2026.1698599

**Published:** 2026-04-13

**Authors:** Werner Alexander Isop

**Affiliations:** Independent Researcher, Graz, Austria

**Keywords:** AI systems, blended learning, extended reality, human-centric, Open Auditorium, robot operating system

## Abstract

Growing interest in extended reality and artificial intelligence (AI) over the past decade has led to the development of a variety of powerful methods with considerable potential for blended learning. However, with great power comes great responsibility. To date, blended learning tools still struggle with the incorporation of practical regulations, preserving natural human presence in the use of AI systems (AIS). This article introduces Open Auditorium (OA), an extended reality blended learning (XR-BL) tool with human-centric ethical conditions of use (COU). Building on conceptual guidelines from previous studies, OA requires educators and learners to be natural human actors, while AIS assume vital yet strictly supportive roles. Furthermore, OA combines a diverse range of input modalities and interactions from pedagogy, extended reality and AI with blended learning. Designed for researchers, educators, and learners, OA aims to make research, teaching, and learning more interactive and expressive. The software architecture of OA is based on the Robot Operating System (ROS) and open source, providing a modular tool that encourages further community-driven research and development. This technology and code article summarizes key design steps, essential features, hardware and software architecture, and implementation of the tool, including a comparison between selected ethical and unethical use cases. Ultimately, this work could provide a reference for the design and implementation of future similar tools and help decision-makers to facilitate a responsible use of AIS in education.

## 1 Introduction

The long-established interactive process of teaching and learning is complex, multifaceted, and fundamentally human-centric (Crow, 1980; Kansanen, 1999; Xhemajli, 2016; Giorgdze and Dgebuadze, 2017; Senthamarai, 2018; Buehl, 2023; Blyznyuk and Kachak, 2024). Consequently, the essential resulting goals and use cases are centered on the interactive development of the skills and competencies of natural human educators and learners (Zamora and Zamora, 2022). Significant technological progress has enriched the field of education through extended reality (XR) (Fowler, 2015; Soliman et al., 2021; Daling et al., 2022; Meccawy, 2022; Van der Meer et al., 2023; Vázquez-Villegas et al., 2023; Bairaktarova et al., 2023) and assistive AI systems (AIS) (Saeed et al., 2024; He et al., 2024; Singh, 2024). However, this progress has increased the need for a responsible handling of related XR-technologies (Fernández-Batanero et al., 2024; Khlaif et al., 2024), and AIS (Yan et al., 2024; Yelamarthi et al., 2024; Tzirides et al., 2024; Hakim and Wahyuni, 2024). As the capabilities and applications of AIS continue to grow rapidly, natural human actors must continuously develop their digital skills and competencies to keep pace with this progress (Tuomi, 2020; Vitezić and Perić, 2024). In the education domain, for instance, recent studies have introduced methods and approaches to fulfill future requirements for digital skills (Spitzer et al., 2022) and to address sustainable development goals (SDG) (Nasir et al., 2023; Abulibdeh et al., 2024; Khattri et al., 2025). At the same time, alongside advances of assistive AIS, studies focusing on different educational stages have raised concerns about the ethical challenges and implications of their use (Akgun and Greenhow, 2022; Córdova and Vicari, 2022; Nguyen et al., 2023; Yang Z. et al., 2024; Poli et al., 2025; Nnorom, 2025; Alqahtani and Wafula, 2025; Abbasi et al., 2025), particularly for higher education (Rane et al., 2025).

To broadly counteract misinterpretations and misuse of AIS in the European Union (EU), a robust high-level regulatory framework has been established in recent years (European-Commission-AI-HLEG, 2019, 2021; European-Commission, 2021, 2023; European-Parliament, 2024). More detailed guidelines for everyday teaching and learning have been created by the The European Commission (Tuomi, 2019; European-Commission-Directorate-General, 2022). Specifically, (Tuomi 2020) advanced a digital ethics pedagogy, merging AI ethics literacy and teacher competency frameworks (Ilkka, 2018; Tuomi, 2019), whereas in the United States more recent frameworks also emphasize teaching with AI. However, with the AIS acting in a co-learner role, instead of in a replacing (Bowen and Watson, 2025). The focus is on developing the (digital) skills and competencies of educators and learners and raise awareness of the ethical use of AIS (Crawford et al., 2023; Kooli, 2023; Qadir, 2023; Burgue lopez, 2024; Friesel et al., 2024; Usher and Barak, 2024; Leon et al., 2025). Consequently, related research merging the fields of blended learning (BL), extended reality (XR), and AI reflects an increased awareness of ethical implications (Rangel-de Lazaro and Duart, 2023; Iqbal et al., 2023; Kuleto et al., 2023; Memarian and Doleck, 2024). Nevertheless, these tools lack detailed specifications of low-level properties, essential for preserving natural human presence in AIS-assisted educational scenarios (Fischer, 2022; Pagano et al., 2024; Isop, 2025). In this regard, supporting ethical principles from a professional teaching perspective are extensively addressed by (Rogers 1969) and stress the strict relevance of natural human presence to foster empathy and trust during teaching and learning (Valett, 1977; Rogers and Freiberg, 1994). Furthermore, to date, no related tool suggests a detailed design summary, including essential activities, goals, and use cases with a reference to current high-level regulations and reflections on potential misuse. In addition, clear guidance on responsibly sharing presence between natural human actors and AIS, based on a concrete set of ethical conditions of use (COU) for the interactive process of teaching and learning, remains absent. In response, this technology and code-article introduces a human-centric AIS assisted XR-BL tool. The design aims at the responsible use of assistive AIS, shaping an even more interactive and expressive teaching and learning process (Kore, 2022). Furthermore, the design underlines a human-centric approach (Shawe-Taylor and Dignum, 2024) in which natural human researchers, educators, and learners are preserved as the main actors. Summarizing the ethical implications revealed during the design process, the tool could ultimately provide a reference for implementation and help decision-makers facilitate the responsible use of AIS in education.

## 2 Related tools for education

Over the past three decades, research across the different dimensions of XR has explored a rich variety of methods, particularly in the context of education (Huang and Tseng, 2025; Hanid et al., 2025; Fernández-Cerero et al., 2025). Derived from the reality–virtuality (RV) continuum (Milgram et al., 1995a), XR describes the gradual transition from the visually real to environments with increasing amounts of virtual content. Once projected onto displays (Milgram et al., 1995b), XR serves as an umbrella term encompassing various sub-disciplines in visualization, whilst XREd particularly addresses educational applications (McDonnell et al., 2024; Memarian and Doleck, 2024; Lampropoulos and Chen, 2025). XR includes augmented reality (AR) (Avila-Garzon et al., 2021; Koumpouros, 2024; Dhaas, 2024), mixed reality (MR) (Shaytura et al., 2021; Suryodiningrat et al., 2024) and virtual reality (VR) (Rojas-Sánchez et al., 2023; Yang C. et al., 2024), all of which have gained in popularity since the turn of the millennium, particularly in engineering education (Suhail et al., 2024; Antoine et al., 2024; Shah et al., 2024). With an emphasis on virtual reality (VR) and 3D virtual learning environments (VLE), related educational tools (Kavanagh et al., 2017; Khukalenko et al., 2022; Han and Bailenson, 2024; Gower et al., 2025) cover a wide range of (immmersive) “virtual classrooms” (Rizzo et al., 2000, 2009; Sharma et al., 2013; Dong, 2016; Liou and Chang, 2018; Chatwattana et al., 2020; Gao et al., 2021; Liu et al., 2021; Hedrick et al., 2022; Liu et al., 2022), as well as “virtual universities” (Chau et al., 2013; Chatwattana et al., 2023; Wang et al., 2024). However, besides of that they utilize purely virtual avatars, these approaches have not addressed a seamless human-centric integration of assistive AIS, XR, and BL.

### 2.1 Extended reality blended learning tools

The application of XR tools to enrich the interactive process of teaching and learning has been intensively investigated, with solutions focusing on AR (Dengel et al., 2022), MR (Nebeling et al., 2020; Hu et al., 2024), and VR (Choi et al., 2016; Verkuyl et al., 2022). Over the past two decades, the educational spectrum of XR has gradually expanded from early classic AR tools that replaced conventional desktop experiences (Billinghurst et al., 2001; Schmalstieg et al., 2002; Kaufmann and Meyer, 2008; Ivanova et al., 2014; Kovalenko et al., 2021), through related notable milestones in MR (Kirkley and Kirkley, 2005; Quint et al., 2015; Steed et al., 2022) and VR (Hughes and Stapleton, 2005; Getso and Bakon, 2017; Díaz et al., 2020; Antonelli et al., 2023), to more recent tools situated within the broader context of XR (Herur-Raman et al., 2021; Liarokapis et al., 2024b,a). The essential goal is to interactively extend the visual perceptions of educators and learners, resulting in a more immersive, expressive, and lively user experience. With an increasing emphasis on collaboration, recent XR-driven educational tools incorporate online, remote, telepresence, and networked teaching and learning within 3D virtual learning environments (VLEs) (Ali et al., 2019; Fadzli et al., 2020; Mourtzis and Angelopoulos, 2023; Pagani et al., 2023; Picard et al., 2024) and can be collectively described as extended reality-blended learning tools (XR-BL) tools (Kumar et al., 2021; Pringle et al., 2022; Fortman and Quintana, 2023; Burnett, 2024; Crogman et al., 2025).

### 2.2 AIS-assisted tools

Recently, research has shifted toward augmenting established XR and BL methods with the rapidly evolving capabilities of AIS (Wadams and Schick-Makaroff, 2022; Alshahrani, 2023; Pratama et al., 2023; Murali et al., 2024). Non-embodied AIS have been utilized to enhance teaching and learning by creating content and instructions (Linares-Pellicer et al., 2024; Kumar et al., 2024; Xu, 2025), fostering student performance (Ciolacu et al., 2020; Hamadneh et al., 2022; Nguyen et al., 2025), and streamlining educational logistics (Ait Ounejjar et al., 2024). Other studies have recognized the value of virtually embodied AIS (Kim et al., 2018; Richter, 2024; Paolo et al., 2024; Chitra and Saleem Raja, 2025; Yang et al., 2025), while recent studies have explored pedagogical and social AIS in collaborative XR environments (Grivokostopoulou et al., 2020; FUNDACIO-EURECAT, 2022a,b; Ihara et al., 2023; Liarokapis et al., 2024b; Chheang et al., 2024). Notably, such AIS are not necessarily represented human-like, but with alternative representations, such as real flying robots (Kim et al., 2016; Al Khawaldah et al., 2025), or simulated ones (Karjalainen et al., 2017; Tazir et al., 2023). The goal is to improve interactions with educators and learners through the perceived embodied presence of the AIS and its nonverbal behavior.

### 2.3 Closest related tools

**Figure 1:**
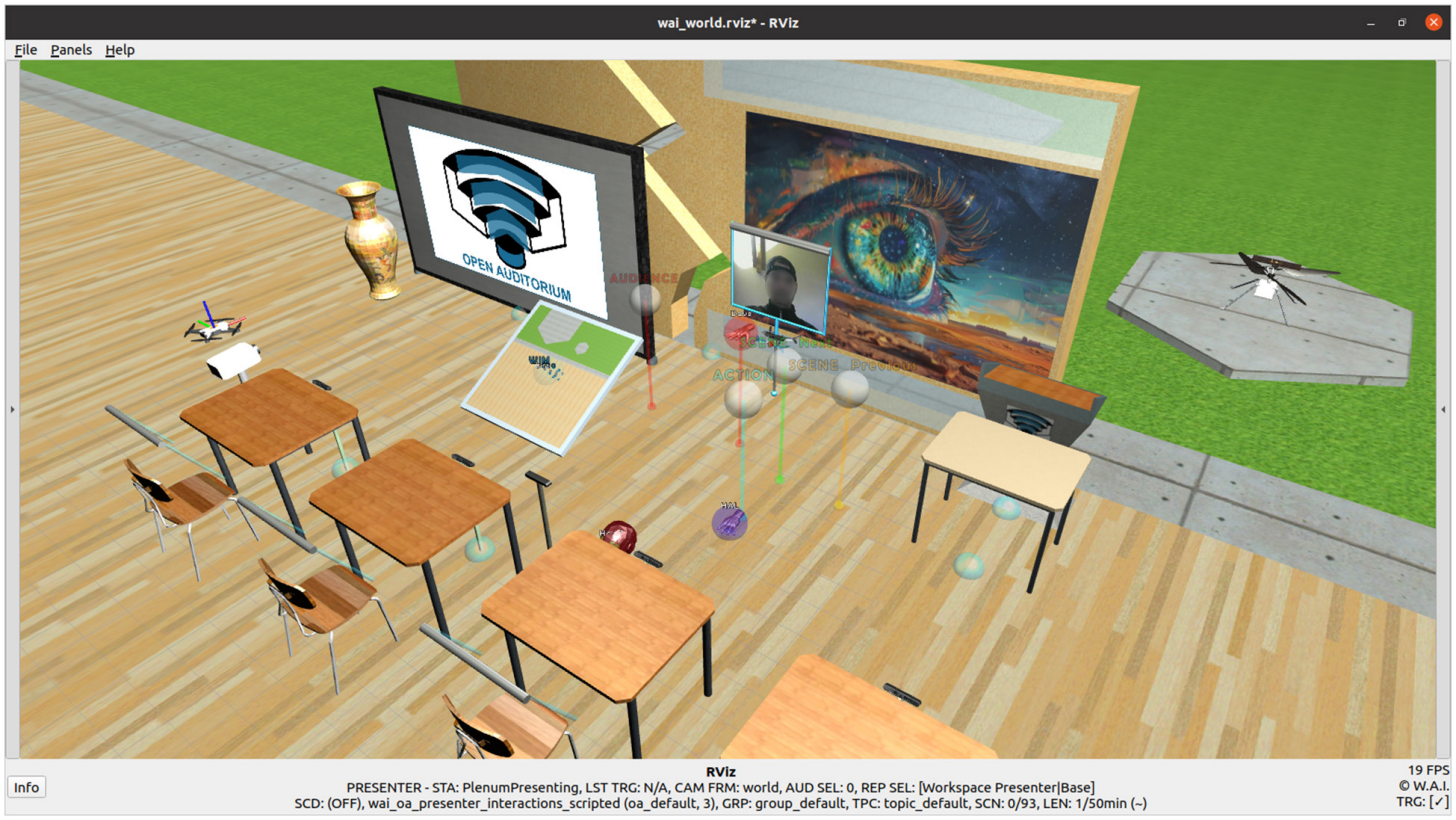
Typical representation of OA in RViz (Kam et al., 2015) with its XR environment, virtualized natural human actors, and MARVIN the display drone companion, acting as assistive AIS. In the background, an interactive virtual reassembly of a Mars Helicopter is prepared for experimentation (Tzanetos et al., 2022; NASA, 2024)^1^.

Similar to other recent XR-BL tools (Liarokapis et al., 2024b; Chheang et al., 2024) that focus on empirical validation of AI-assistance (Gu et al., 2024; Lee et al., 2025; Fajri et al., 2025), Open Auditorium (OA) pursues the shared goal of enriching the overall teaching and learning experience, rendering it more immersive, interactive, and lively (Figure 1). Furthermore, it aims to provide a better understanding of content in both individual and collaborative settings. OA utilizes the basic principles of constructivism (Juvova et al., 2015; Zajda and Zajda, 2021; Alpaydın and Demirli, 2022; Mishra, 2023) for the design of its teaching and learning sessions as well as its interactions (Isop, 2024). Regarding AIS assistance, a variety of related tools use embodied real (Al Khawaldah et al., 2025) or virtualized representations of AIS with natural language processing (NLP) interfaces in XR environments (Huang et al., 2019; Baytas et al., 2019; Tazir et al., 2023). In a similar manner, OA introduces MARVIN, a purely virtual display drone companion (Scheible et al., 2013; Nozaki, 2014; Scheible et al., 2017). Equipped with a NLP interface, a 2D-display on its shoulders, and a 3D-projector, MARVIN acts as an assistive AIS. Regarding ethical concerns, the goal is to responsibly enrich the interactive process of teaching and learning. In this respect, studies have highlighted concerns about the ethical use of AIS in research and education (Sijing and Lan, 2018; Akgun and Greenhow, 2022; Kassymova et al., 2023; Nguyen et al., 2023; Salloum, 2024), whilst global regulators have been addressing these issues with increasingly sophisticated high-level frameworks (European-Commission, 2025). Nevertheless, existing work does not consider low-level properties to in-detail regulate the presence of actors in typical educational settings-essential for preserving human-centric tools (Isop, 2025). To date, no AIS-assisted XR-BL tool has proposed a concrete set of human-centric ethical COU to sustainably preserve natural human presence in the use of AIS in education (Table 1).

**Table 1:** The ethical conditions of use of Open Auditorium (OA).

EU requirements	Properties	Conditions of use
		The use of OA in *education* during the *interactive process of teaching and learning* to achieve the *development of skills and competencies* is permitted, provided that the following conditions are met:
HAO, ACC	1) Role	Roles of all actors (educators, learners, and AIS) must be clearly defined. Actors can either assume leading, guiding, participating, or supporting roles. Respecting fundamental human rights, all use cases to develop skills and competencies, during the interactive process of teaching and learning, are essentially conducted by natural human actors. Educators are leading or guiding, learners are leading or participating. AIS are supporting, at different IOL-according to the educational stage; however, educators or learners are not to be replaced (Figure 8a).
HAO, ACC	2) Multiplicity	If possible, and not intended by the teaching/learning activity otherwise (e.g., homework), the interactive process of teaching and learning must involve *N*(1..*) natural human educators and *n*(1..*) natural human learners (at different IOL—according to the educational stage), and *m*(0..*) AIS (Figure 8a).
SEW	3) Behavior	If not intended by the teaching/learning activity otherwise (e.g, “learn from failure”), all actors must always promote (maintain, facilitate, or encourage) ethical behavior during the interactive process of teaching and learning.
SEW	4.1) Synchron.	If possible (e.g., not restricted due to crisis), and not intended by the teaching/learning activity otherwise (e.g., “out-of-class” flipped classroom), the interactive process of teaching and learning must be conducted synchronously, at different IOL – based on the educational stage. Predominant asynchronous scenarios (e.g, pure offline prerecording of teaching/learning sessions and simple replaying) may be indicated (e.g., by distinct labels/colors, decreased quality of visual representation, etc.) or avoided in general (Figure 8b).
SEW	4.2) Location	If possible (e.g., not restricted due to crisis), and not intended by the teaching/learning activity otherwise (e.g., “homework”), the interactive process of teaching and learning must be conducted colocated, at different IOL - according to the educational stage. Predominant dislocated scenarios may be conducted with respect to condition 4.1).
HAO, TRA, ACC	4.3) Embod.	All actors must be, at any time, embodied. Natural human educators and learners must preserve visual coherence of their embodiment, at different IOL-according to the educational stage. AIS must be visually embodied and clearly labeled as such, including an indication of their system boundaries (location) to avoid omnipresence and to increase transparency and trust (Figure 8c).
HAO, TRA, ACC	4) Vis. Rep.	All natural human actors are visually represented as real or, at least, by their virtualization, at different IOL - according to the educational stage. AIS may be represented purely virtual at any time; however, they must not mimic natural human actors (Figure 8c).

## 3 Tool design

**Figure 2:**
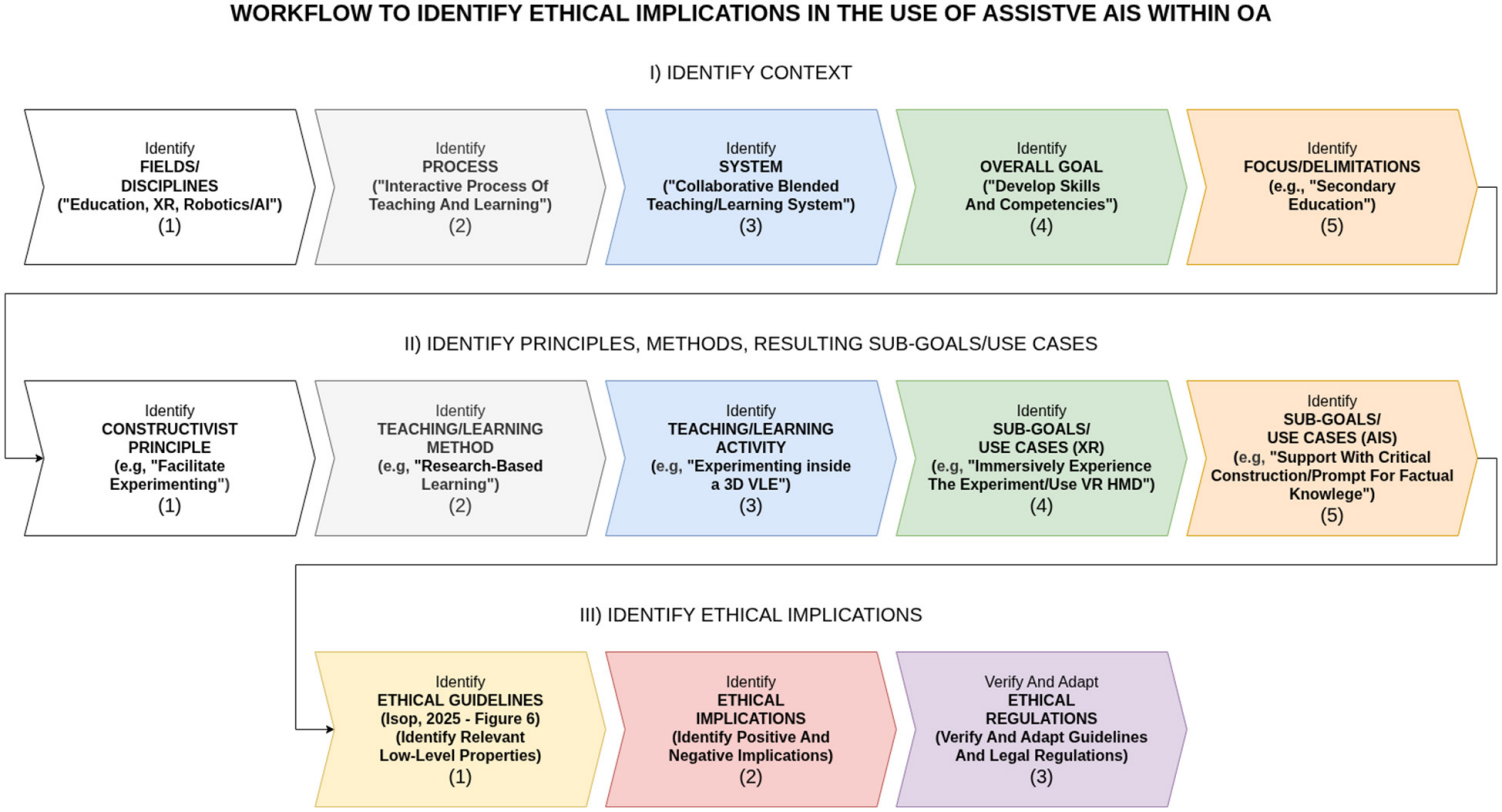
Workflow of the design summary to identify, context (I.1 to I.5), concrete sub-goals/use cases (II.1 to II.5), and potential ethical implications (III.1 to III.3) in the use of assistive AIS within OA.

**Figure 3:**
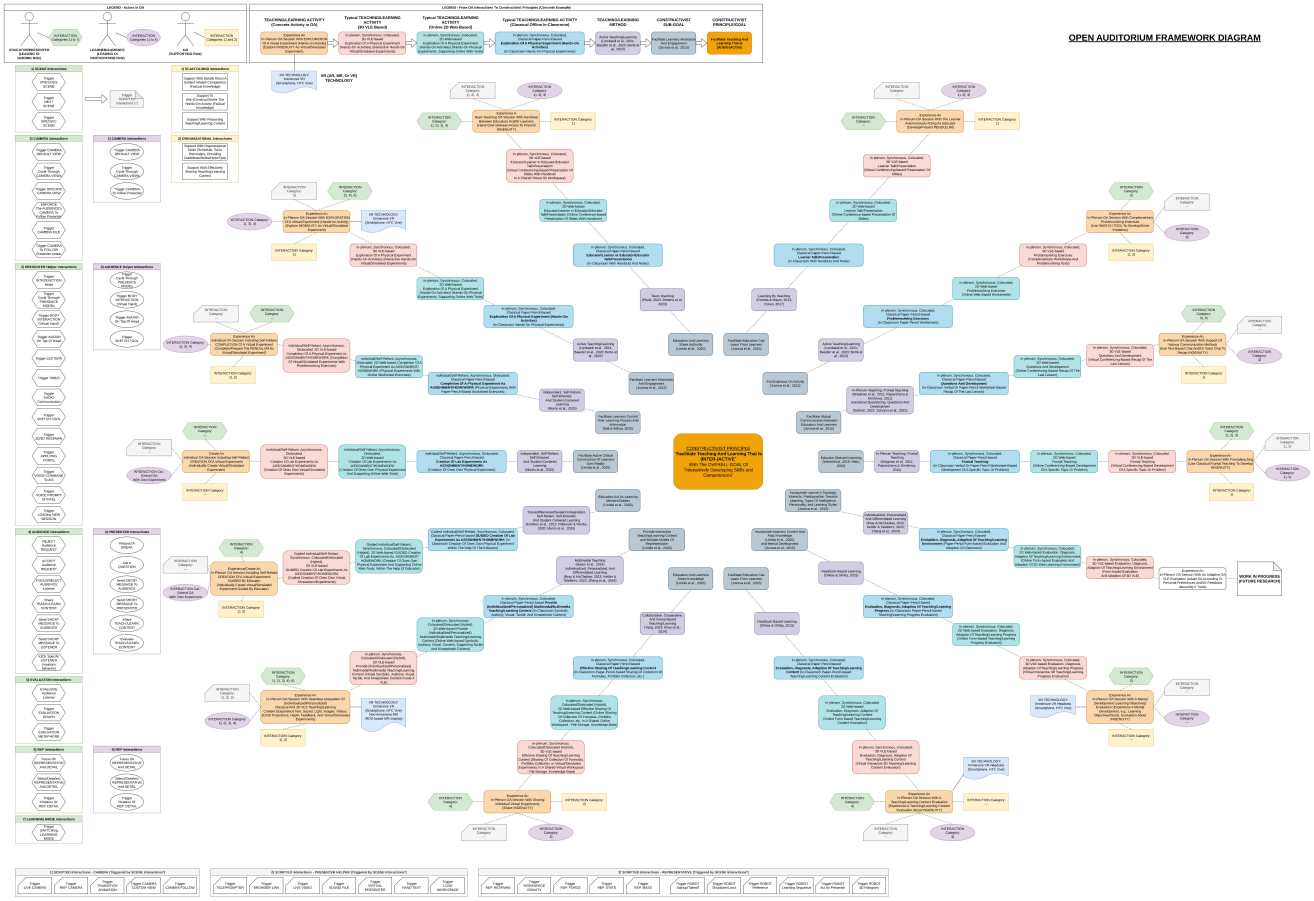
Framework diagram of actors/roles, interactions, resulting constructivist principles, and goals to “Facilitate teaching and learning that is (INTER-)ACTIVE” with focus on engineering education at secondary level.

**Figure 4:**
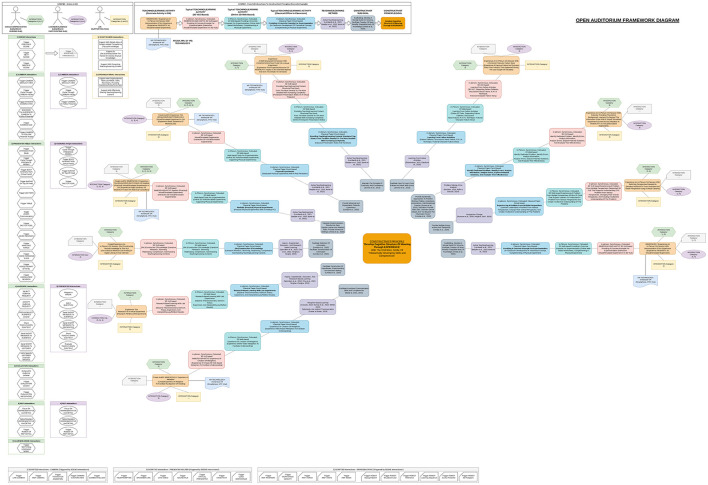
Framework diagram of actors/roles, interactions, resulting constructivist principles, and goals to “Develop A Cognitive Structure Of Meaning Through EXPERIENCE” with focus on engineering education at secondary level.

**Figure 5:**
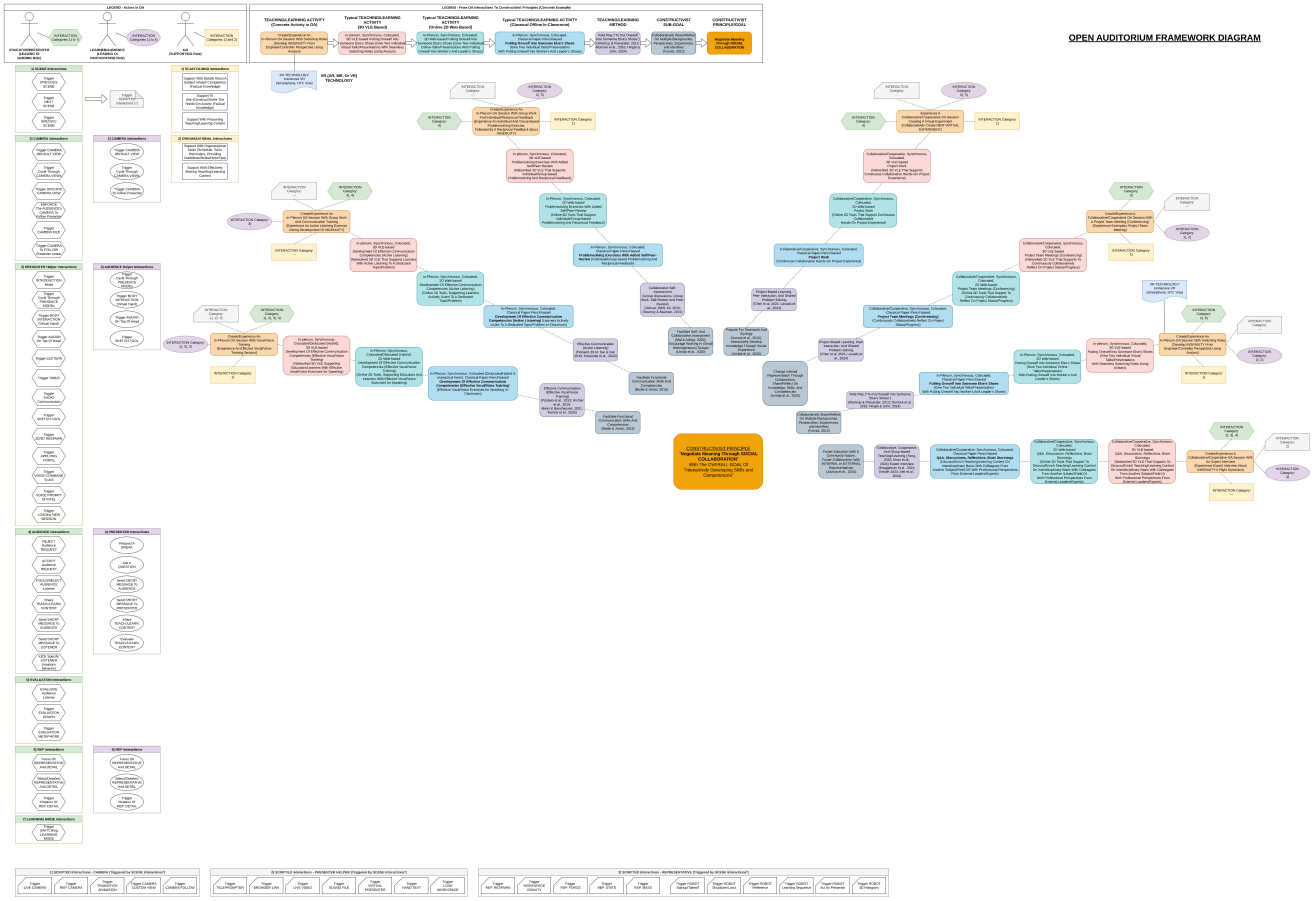
Framework diagram of actors/roles, interactions, resulting constructivist principles, and goals to “Negotiate meaning through SOCIAL COLLABORATION” with focus on engineering education at secondary level.

From the above analysis, the design of state-of-the-art AIS-assisted XR-BL tools requires highly interdisciplinary perspectives, including design principles from robotics (AIS, AI companions, and ethical use), visualization (XR), interaction (HCI and HRI), and pedagogy (constructivism, BL, and collaborative learning). Thus, the design and implementation of such tools is challenging. Considering the field of “education” as an “interactive process of teaching and learning” with the overarching goal of “developing skills and competencies,” principles and methods from pedagogy serve as a solid starting point. Essential principles from social constructivist theory supporting “active learning” (Pardjono, 2016) are well established, widely accepted (Naylor and Keogh, 1999; DeVries, 2004; Richardson, 2005; Mogashoa, 2014; Alanazi, 2016; Kritt and Budwig, 2022; Nurhasnah et al., 2024), and, become even more tangible when translated into concrete practical guidelines (Willis, 2000; Cunningham, 2006; Juvova et al., 2015; Mal and Adhya, 2020; Umida et al., 2020; Umida and Suyunova Aziza, 2020). Due to the rapid pace of digital technology (Venkateswari, 2024), design principles and methods for BL are relatively young, more diverse (Hoic-Bozic et al., 2008; Sun et al., 2017; Cleveland-Innes and Wilton, 2018; Bhadri and Patil, 2022; Janse van Rensburg and Oguttu, 2022; Wadams and Schick-Makaroff, 2022; De Bruijn-Smolders and Prinsen, 2024), and still under investigation (Li et al., 2024; de Souza and Debs, 2024; Chan, 2025). Underlying pedagogical frameworks for digital learning theory development focus on self-directed, technology-mediated (Knowles, 1984), and lifelong learning (Blaschke, 2012), whereas critical perspectives regarding the use of AIS are extensively discussed in (Rane et al. 2025). In accordance, methods and applications of XR in education have been extensively researched in recent years (Yang et al., 2020; Rawat et al., 2022; Spitzer et al., 2022; Chang et al., 2023; Samala et al., 2024). Consequently, the design of OA is based on classical principles from pedagogy, in particular constructivism. Essentially, OA investigates the gradual technological extension toward online learning (BL), advanced techniques from visualization (XR), and ethical AIS-assistance. In the first step, the core principles and goals for the design of educational environments from social constructivist theory (Honebein, 1996; Tam, 2000; Fosnot, 2013; Bada and Olusegun, 2015; Bodie and Jones, 2015; Mal and Adhya, 2020) are connected to the related teaching/learning methods. For the sake of completeness, also classical plenary and frontal teaching methods have been considered because of their efficiency, low complexity, and prevalence (Bregman et al., 2011; Papancheva and Dimitrova, 2011; Westerholz, 2019; Seitz, 2020). In the second step, the principles and goals are projected onto selected teaching/learning activities in the context of collaborative BL. Third, the activities are extended to concepts from XR environments with a particular focus on 3D VLEs. In the final step, concrete use cases of assistive AIS are derived, including their potential ethical implications and the current status of regulations. The workflow of OA's ethical design is depicted in Figure 2. Complementary framework diagrams underpin the interplay between actors/roles, interactions, and resulting constructivist principles and goals with focus on in-class teaching/learning activities in engineering education at secondary level (Figures 3–5). Related core constructivist principles and associated teaching methods are divided into three major categories and listed as follows.

**I) Facilitate teaching and learning that is (INTER-)ACTIVE**
  1. Put emphasis on activity (Juvova et al., 2015)
    - Play-based learning (Bubikova-Moan et al., 2019; Vidal-Carulla et al., 2021)
    - Active teaching/learning (Lombardi and Shipley, 2021; Baepler et al., 2023; Børte et al., 2023)
  2. Educators act as learning mentors/guides (Umida and Suyunova Aziza, 2020; Umida et al., 2020)
    - Tutored/mentored/guided independent, self-reliant, (R1-C3): [self-directed (Knowles 1984); (Blaschke 2012), and student-centered learning (Kooloos et al., 2012; Robinson and Persky, 2020; Morris et al., 2025)]
    - Flipped classroom (Tucker, 2012; Baig and Yadegaridehkordi, 2023; Schmid et al., 2023)
    - Massive open online course (MOOC) (Stracke and Trisolini, 2021; Moore and Blackmon, 2022; Loh et al., 2024)
  3. Educators and learners share authority (Umida and Suyunova Aziza, 2020; Umida et al., 2020)
    - Team Teaching (Plank, 2023; Roberts et al., 2023)
  4. Educators and learners share knowledge (Umida and Suyunova Aziza, 2020; Umida et al., 2020)
    - Collaborative, cooperative, and group-based teaching/learning (Yang, 2023; Khan et al., 2024)
  5. Facilitate that educators can learn from learners (Juvova et al., 2015)
    - Learning by teaching (Fiorella and Mayer, 2013; Duran, 2017)
    - Feedback-based learning (Ghilay and Ghilay, 2015)
  6. Facilitate mutual communication between educators and learners (Juvova et al., 2015)
    - Intentional Questioning, Questions And Development (Salmon and Barrera, 2021); (Suryana et al., 2021)
    - Active plenary teaching (Friederike, 2015)
  7. Facilitate active critical construction of learners own reality (Umida and Suyunova Aziza, 2020; Umida et al., 2020)
    - Independent, self-reliant, self-directed, and student-centered learning (Morris et al., 2025)
  8. Facilitate learners control over learning process and information (Mal and Adhya, 2020)
    - Montessori method (Kayili and Ari, 2011; Kıran et al., 2021)
    - HighScope approach (Berčnik and Rožman-Krivec, 2024; Weikart, 2024)
    - Independent, self-reliant, self-directed, and student-centered learning (Morris et al., 2025)
    - Flipped classroom (Tucker, 2012; Baig and Yadegaridehkordi, 2023; Schmid et al., 2023)
    - Massive open online courses (MOOCs) (Stracke and Trisolini, 2021; Moore and Blackmon, 2022; Loh et al., 2024)
  9. Facilitate learners motivation and engagement (Juvova et al., 2015)
    - Active teaching/learning (Lombardi and Shipley, 2021; Baepler et al., 2023; Børte et al., 2023)
    - Flipped classroom (Tucker, 2012; Baig and Yadegaridehkordi, 2023; Schmid et al., 2023)
    - Massive open online course (MOOC) (Stracke and Trisolini, 2021; Moore and Blackmon, 2022; Loh et al., 2024)
  10. Provide interactive teaching/learning content, provide multiple modes of representation, trigger as many sensory channels as possible (Umida and Suyunova Aziza, 2020; Umida et al., 2020)
    - Multimodal teaching (Harun and Singh, 2024)
  11. Incorporate learners current and past knowledge (Umida and Suyunova Aziza, 2020; Umida et al., 2020)
    - Feedback-based learning (Ghilay and Ghilay, 2015)
  12. Incorporate learner's mental development (Juvova et al., 2015)
    - Feedback-based learning (Ghilay and Ghilay, 2015)
  13. Incorporate learner's topology, interests, predisposition toward learning, types of intelligence, personality, and learning styles (Juvova et al., 2015)
    - Individualized, personalized, and differentiated learning (Bray and McClaskey, 2012; Kettler and Taliaferro, 2022; Zhang et al., 2024)
**II) Develop A Cognitive Structure Of Meaning Through EXPERIENCE**
  1. Scaffolding, develop a mental model or schema, facilitate creation of most-readily graspable body of knowledge (Umida and Suyunova Aziza, 2020; Umida et al., 2020)
    - Play-based learning (Bubikova-Moan et al., 2019; Vidal-Carulla et al., 2021)
    - Active teaching/learning (Lombardi and Shipley, 2021; Baepler et al., 2023; Børte et al., 2023)
  2. Provide multiple perspectives and viewpoints (Umida and Suyunova Aziza, 2020; Umida et al., 2020)
    - Perspective Change (Rumore et al., 2016; Hingle and Johri, 2024)
  3. Provide systematic approaches to problem solving, finding connections, associations, interdisciplinary transfer (Juvova et al., 2015)
    - Problem-solving, error analysis (Rushton, 2018; Arifin and Bonyah, 2024)
  4. Educators provide main idea, learners derive details and “go beyond the information given” (Umida and Suyunova Aziza, 2020; Umida et al., 2020)
    - Problem-solving, error analysis (Rushton, 2018; Arifin and Bonyah, 2024)
  5. Facilitate how to learn from failure and work with errors (Juvova et al., 2015)
    - Learning from failure activities (Edmondson, 2011; Darabi et al., 2018)
  6. Maintain the principle of continuity and consistency (Juvova et al., 2015)
    - Active teaching/learning (Lombardi and Shipley, 2021; Baepler et al., 2023; Børte et al., 2023)
  7. Provide meaning and organization toward experience (Umida and Suyunova Aziza, 2020); (Umida et al., 2020)
    - Active teaching/learning (Lombardi and Shipley, 2021; Baepler et al., 2023; Børte et al., 2023)
  8. Integrate experimentation directly into task, situate learner into realistic tasks, provide immersive settings (Umida and Suyunova Aziza, 2020; Umida et al., 2020)
    - Active Teaching/Learning (Lombardi and Shipley, 2021; Baepler et al., 2023; Børte et al., 2023)
  9. Facilitate selection of information (Umida and Suyunova Aziza, 2020; Umida et al., 2020)
    - Inquiry-, experiential-, discovery-, and research-based learning (Spronken-Smith and Walker, 2010; Chu et al., 2021; Singha and Singha, 2024)
  10. Facilitate transformation of information (Umida and Suyunova Aziza, 2020; Umida et al., 2020)
    - Inquiry-, experiential-, discovery-, and research-based learning (Spronken-Smith and Walker, 2010; Chu et al., 2021; Singha and Singha, 2024)
  11. Facilitate construction of hypotheses, experimenting, and decision making (Umida and Suyunova Aziza, 2020; Umida et al., 2020)
    - Reggio Emilia approach (Inan et al., 2010; Gencer and Gonen, 2015)
    - Inquiry-, experiential-, discovery-, and research-based learning (Spronken-Smith and Walker, 2010; Chu et al., 2021; Singha and Singha, 2024)
  12. Facilitate functional communication skills and competencies (Bodie and Jones, 2015)
    - Metaphor-based learning (Cunsolo et al., 2010; Murray-Rust et al., 2022; Weller, 2022)
    - Selectively use indirect communication (Baldacchino and Herner, 2024)
**III) Negotiate meaning through SOCIAL COLLABORATION**
  1. Foster education with a community nature, foster collaboration with INTERNAL or EXTERNAL representatives (Juvova et al., 2015)
    - Collaborative, cooperative, and group-based teaching/learning (Yang, 2023; Khan et al., 2024)
    - Expert interview (Bruggeman et al., 2021; Kiesler, 2023; Aler Tubella et al., 2024)
  2. Collaboratively share/reflect on multiple backgrounds, perspectives, experiences, and identities (Fosnot, 2013)
    - Play-based learning (Bubikova-Moan et al., 2019; Vidal-Carulla et al., 2021)
    - Role play (“to put oneself into someone else's shoes”) (Westrup and Planander, 2013; Rumore et al., 2016; Hingle and Johri, 2024)
  3. Change internal representation through collaboration, share/reflect on knowledge, skills, and competencies (Umida and Suyunova Aziza, 2020); (Umida et al., 2020)
    - Project-based learning, peer interaction, and shared problem-solving (Chen et al., 2021; Lavado-Anguera et al., 2024)
  4. Preparation for teamwork and synergy (Juvova et al., 2015)
    - Reggio Emilia approach (Inan et al., 2010; Gencer and Gonen, 2015)
    - Project-based learning, peer interaction, and shared problem-solving (Chen et al., 2021; Lavado-Anguera et al., 2024)
  5. Interactively develop knowledge through social experience (Umida and Suyunova Aziza, 2020; Umida et al., 2020)
    - Reggio Emilia approach (Inan et al., 2010; Gencer and Gonen, 2015)
    - Developing and promoting social and emotional competencies (Alwaely et al., 2021; Crowell, 2021; Fatahi et al., 2023; Zinsser et al., 2023)
    - Project-based learning, peer interaction, and shared problem-solving (Chen et al., 2021; Lavado-Anguera et al., 2024)
  6. Facilitate self- and collaborative assessment (Mal and Adhya, 2020)
    - Collaborative self-assessment (group discussions, group work, self-review and peer-review) (Vidmar, 2005; Ali, 2015; Flournoy and Bauman, 2021)
  7. Encourage working in (small heterogeneous) groups (Umida and Suyunova Aziza, 2020; Umida et al., 2020)
    - Collaborative self-assessment (group discussions, group work, self-review and peer-review) (Vidmar, 2005; Ali, 2015; Flournoy and Bauman, 2021)
  8. Facilitate functional communication skills and competencies (Bodie and Jones, 2015)
    - Effective communication (active listening) (Howard, 2014; Bodie and Jones, 2015; Dar and Dar, 2019)
    - Effective communication (effective vocal/voice training) (Pizolato et al., 2013; Richter et al., 2016; Meier and Beushausen, 2021; Ramos et al., 2025)

The listed constructivist principles, goals, and associated teaching/learning methods implicate a rich set of administrative and non-administrative activities and related use cases of different actors “in-class” already. Additionally, the interactive process of teaching and learning involves many more “out-of-class” use cases beyond the classroom or lecture hall. Facing recent technological advancements in XR and AI, an additional distinction between the involved actors, synchronous/asynchronous, real/virtual, colocated/dislocated, or hybrid blended learning settings is crucial to define natural human presence. As a result, in detail describing field, process, system, actors and roles, goals and subgoals, and concrete use cases becomes overly complex, but inevitable to properly identify potential ethical implications. Comprehensive and well-structured educational system overviews are available from the European Commission (European-Commission-Eurydice, 2025); however, they remain at a high-level perspective and lack a detailed list of use cases. Other studies concentrate on more detailed system descriptions within BL, however focus on specific educational levels, for example, early childhood education in preschools (Phajane, 2014; Franzén and Hjalmarsson, 2021; Kunt and Avcı, 2023; US Bureau of Labor-Statistics, 2025b; Ghazali et al., 2025), primary education in primary schools (Dean, 2002; Forrester, 2005; Uibu and Kikas, 2008), secondary education in lower- and upper- secondary schools (Wasburn-Moses, 2005; Todorescu et al., 2015; Universiteit-Utrecht, 2025; US Bureau of Labor-Statistics, 2025a; Shohel et al., 2022), and post-secondary/higher/further/tertiary education in high schools or (technical) colleges (Fry et al., 2008; Marshall, 2016; Mohammed and Kassem, 2020; Hoidn and Klemenčič, 2021; Istenič, 2024) and universities (Rhode, 2001; García-Gallego et al., 2015; Donoghue, 2018; Pace et al., 2021; Johnson and Griffin, 2024).

Consequently, based on the conceptual ethical framework laid down in previous works (Isop, 2025), a design summary with a detailed list of uses cases suitable for all educational levels, was crafted during the development of OA. As development is still ongoing, the design summary of OA is provided online as Google Sheet.^4^. It is currently limited to administrative and non-administrative “in-class” activities and related use cases during teaching/learning sessions; however, extension with administrative “out-of-class” use cases, beyond teaching/learning sessions, is planned as future work.

### 3.1 Overview and core concept

The design of OA is centered on a classical networked 3D virtual learning environment (VLE), implemented in ROS (Quigley et al., 2009), and visualized in RViz (Kam et al., 2015). The underlying BL system is distributed among multiple nodes, plugins, and ROS-tools. The involved actors are educators, learners and AIS, and they may interact with the system as presenters or audience (listeners). Teaching and learning in OA is divided into typical teaching/learning sessions, sub-divided into scenes. To ensure ease of use and because of its popularity, each session is organized with common presentation slides. Scene transitions are triggered by the interactions of the presenter, whereas between transitions, all actors can freely move inside the VLE. Various input devices are available for interaction, whilst, in the context of XR, multiple types of displays are enabled. Ranging from classical 2D computer displays to ROS-built-in AR/MR displays and immersive VR displays, OA implements an XR learning environment. The learning environment of OA is purely virtual, ethically representing virtualized presenters (educators), a virtualized audience (learners), and purely virtual assistive AIS for experimentation.

### 3.2 Enhancing interactivity

**Figure 6:**
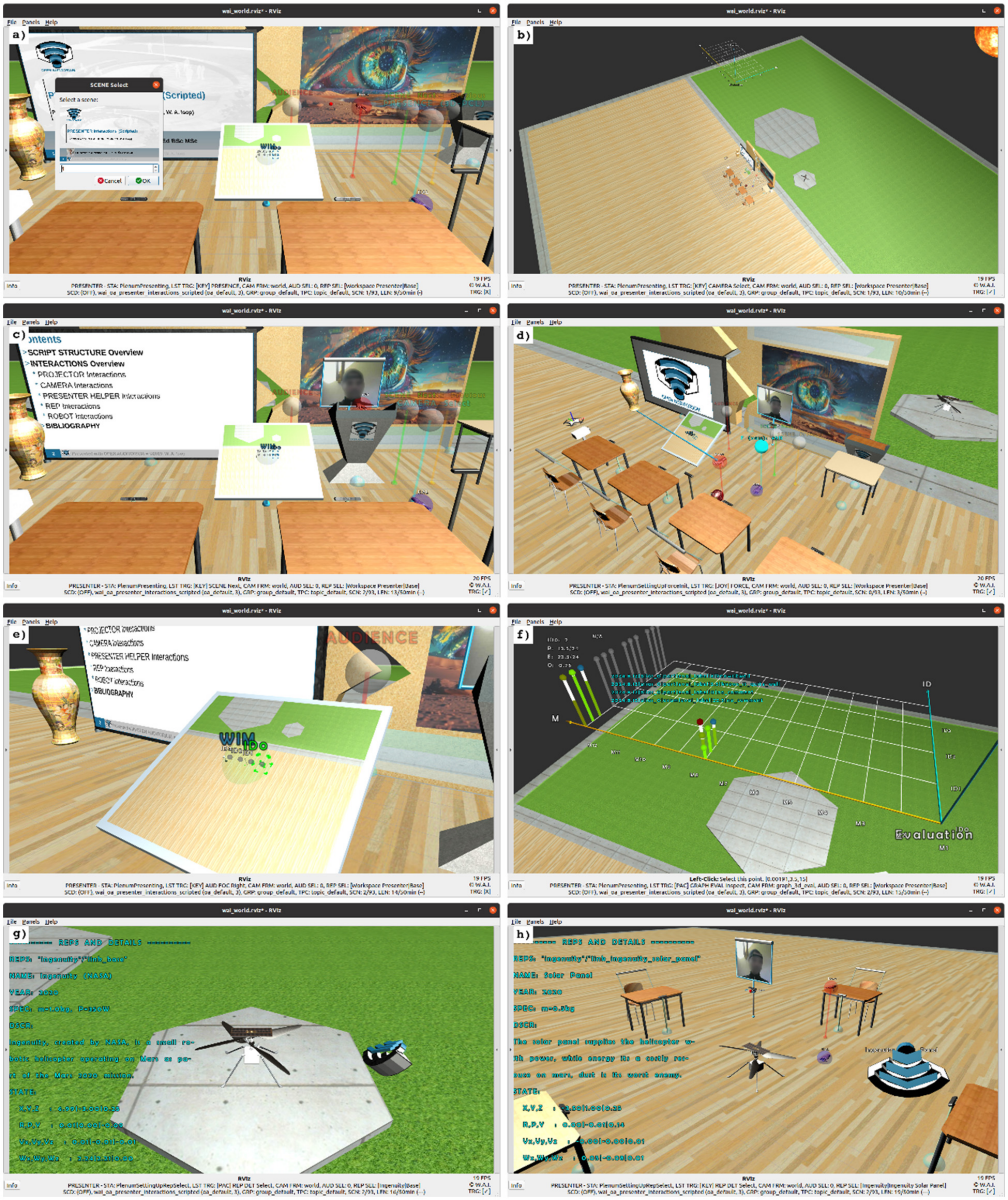
Selected exemplary interactions of the OA Presenter, according to the outline in Section 3.2. The OA Presenter **(a)** selects a specific scene. **(b)** Selects a specific camera view. **(c)** Toggles a virtual lectern. **(d)** Toggles a body interaction to apply a force. **(e)** Focuses on a specific listener from the audience. **(f)** Evaluates a selected listener from the audience. **(g)** Selects a virtual robot to display its details. **(h)** Seamlessly switches to a cooperative learning mode.

To “Facilitate teaching and learning that is (INTER-)ACTIVE” (Section 3, I), OA enables a variety of input devices, ranging from traditional mouse, keyboard, and joypad devices to more advanced devices, such as smartphone, VR-HMD controllers, 3D-sensors (inputs via body-gestures), and a NLP interface. Based on the input devices, educators and learners are provided with a rich set of multimodal interactions divided into different categories. The selected exemplary interactions and their relevant design concepts from XR are listed as follows:

1. **OA Presenter interactions** (also depicted in Figure 6)
  a) SCENE interactions (e.g., “Select a specific SCENE”) - XR scenes (Gomes et al., 2020; Nebeling et al., 2020; Meccawy, 2022; Nebeling, 2022; Chen et al., 2025), virtual projections (Virtual Beamer) (Bischoff, 2004)
  b) CAMERA interactions (e.g., “Transition the CAMERA to an overview”) - Virtual Camera (Christie et al., 2008; Boy et al., 2010)
  c) PRESENTER HELPER interactions (e.g., “Toggle LECTERN in front of presenter”) (Kunz et al., 2002)
  d) PRESENTER HELPER interactions (e.g., “Toggle the BODY INTERACTION of the presenter”) - Virtual hand pointer applying a force (Duval and Fleury, 2009; Mendes et al., 2019)
  e) AUDIENCE interactions (e.g., “FOCUS on specific Audience/Listener”) - World In Miniature (WIM) (Stoakley et al., 1995; Danyluk et al., 2021)
  f) EVALUATION interactions (e.g., “Evaluate EXAMINATION of currently focused Audience/Listener ID with CUSTOM SCORE of predefined fraction”)
  g) REPRESENTATIVE interactions (e.g., “SELECT specific representative and detail”)
  h) LEARNING MODE interactions (e.g., “Switch LEARNING MODE to Cooperative”)
2. **OA Audience (OA Listener) interactions**
  i) CAMERA interactions (e.g, “Transition the CAMERA to CYCLE to the next view in a list of predefined views”) - Virtual Camera (Christie et al., 2008; Boy et al., 2010)
  j) LISTENER HELPER interactions (e.g, “Toggle AVATAR on top of the AUDIENCE/LISTENER's head”) - XR Avatars (Pereira et al., 2019)
  k) PRESENTER interactions (e.g, “Send REQUEST for a BREAK of the AUDIENCE/LISTENER to the presenter”)

### 3.3 Enhancing expressiveness

**Figure 7:**
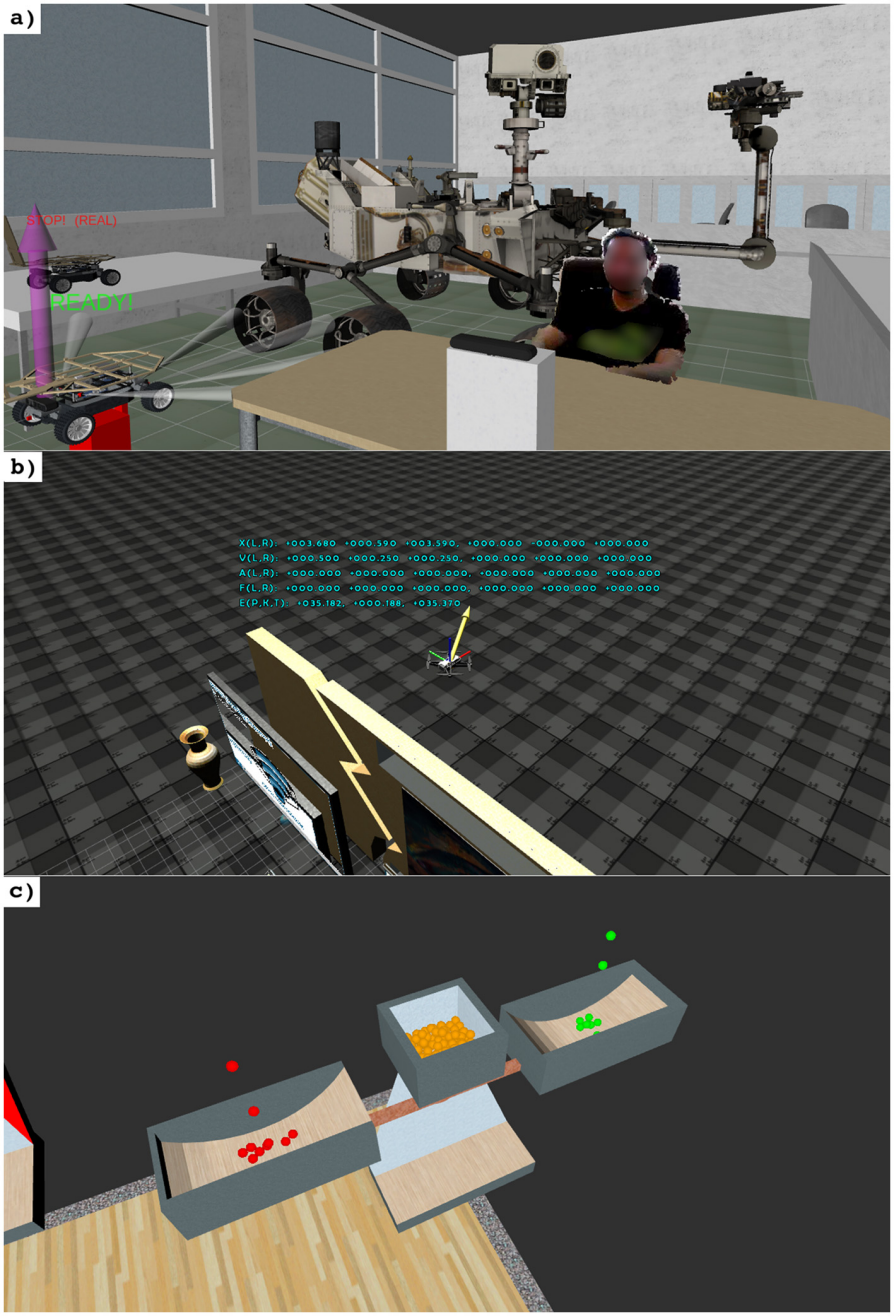
Overview on mechanisms in OA to enhance expressiveness. **(a)** An interactive virtual reassembly of a Mars Exploration Rover is prepared for experimentation (Welch et al., 2013; NASA, 2025). **(b)** Motion of a small sized aerial vehicle, displaying essential physical quantities as part of the learning sequencer. **(c)** A weight balance as grading metaphor, indicating overall (orange), achieved (green) and non-achieved (red) points.

Following the core principles and goals of constructivist theory (Section 3), OA implements various mechanisms to more clearly explain complex connections, problems, and experiments. While further extensions are planned as part of future work, the overarching goal is to “Develop a cognitive structure of meaning through EXPERIENCE” (Section 3, II). OA currently provides “virtual/simulated experiments” (Noor and Wasfy, 2001; Winsberg, 2003; Wörner et al., 2022; Shen et al., 2024) (Section 3, II.1, II.7, II.8, II.11) - to experiment with objects, such as a pendulum or robots (Figure 7a), a “learning sequencer” (Coffey et al., 2012) (Section 3, II.2, II.6, II.7, II.8)—to better understand physics (poses, velocities, accelerations, forces, and energy) of selected experiments up to secondary education (Figure 7b), and “metaphors” (Herrera-Granda et al., 2024) (Section 3, II.12)—to enhance the overall expressiveness of the teaching/learning process (Figure 7c).

### 3.4 Preserving natural human presence in the use of AI systems

**Figure 8:**
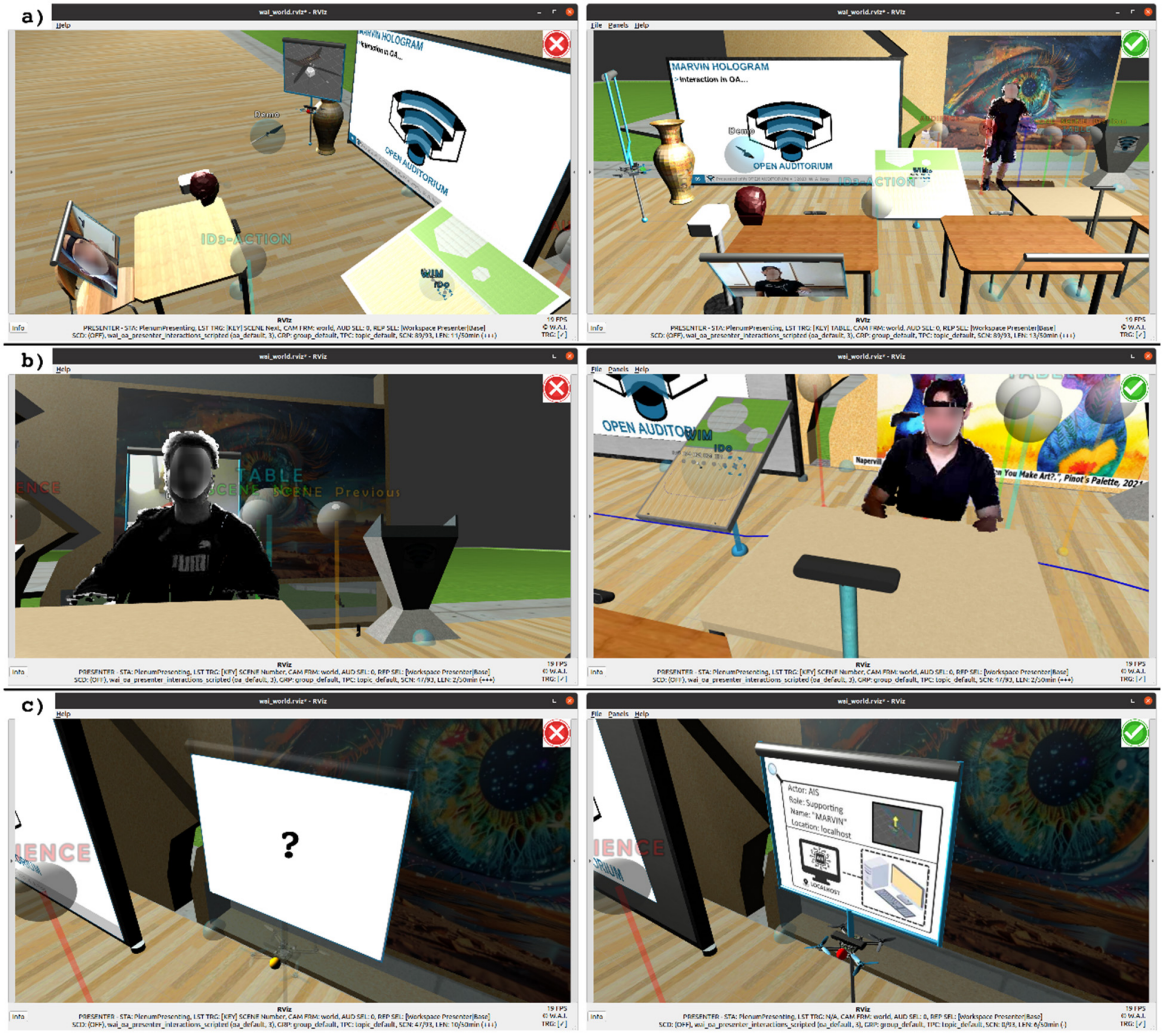
Comparison of ethical (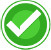) and unethical (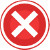) use cases in OA, addressing the role, multiplicity, and visual representation of actors. **(a)** MARVIN is fully replacing a natural human educator. **(b)** The interactive process of teaching and learning, based on a purely virtual presenter, in a predominantly asynchronous setting. **(c)** MARVIN acting as AIS without indication of embodiment, role, and system boundaries.^2^

A core design principle of OA is to preserve the natural human presence, with AIS assigned vital but clearly defined supporting roles that responsibly enhance the interactive process of teaching and learning. The importance of preserving natural human presence is extensively emphasized in humanistic educational frameworks (Rogers, 1969; Valett, 1977; Rogers and Freiberg, 1994), whereas from a professional pedagogical perspective it is fundamental to fostering empathy and trust of learners. To achieve this important human-centric goal, current high-level requirements (European-Commission-AI-HLEG, 2021) and legal regulations (European-Commission, 2021) of the European Union serve as a solid basis, whereas additional low-level properties, to define concrete use cases, were suggested in previous studies (Isop, 2025). Essential drawbacks of this conceptual ethical framework were an extensive design overhead, caused by detailing all typical use cases and identifying potential ethical implications for a specific process and its underlying goal. Moreover, the conceptual framework lacked flexible differentiation for different educational levels. As this current lack became apparent during the design of OA^4^, an additional low-level property was found to be vital to identify ethical implications. Considering typical use cases in education, the low-level property suggests to determine the intensity of AIS use over time during teaching and learning (Von Garrel and Mayer, 2023; McElheran et al., 2024). It is defined with the intensity of use (time the AIS are used during a teaching/learning session, compared to the overall session time) and the occurrence (usage of the AIS in a certain number of teaching/learning sessions, compared to the overall number of available sessions). With the resulting Intensity/Occurrence Level (IOL) it is possible to differentiate between specific intensities of AIS use for different educational levels. For example, in early childhood and primary education, face-to-face interactions between natural human actors, without the need for extensive AIS assistance, may be preferred, as they are vital for the social development of learners (Kurian, 2025). However, with growing independence and sense of responsibility of the learners up to tertiary education a gradual increase of the IOL may foster the development of a symbiosis between social and digital skills. Closely linked to the principle of fairness in a social context (SEW, ACC), the IOLs may enable a trade-off between a very limited, however low-risk, use of AIS, and an ubiquitous, however potentially deteriorating, high-risk exposure to AIS in all areas of education (European-Commission, 2021). Noteworthy, the concrete IOLs in the design summary of OA (see text footnote 4) were preliminarily chosen and are suggested as a basis for discussion. In summary, respecting the role, behavior, multiplicity, visual representation, and IOL of actors is essential to preserve natural human presence in the use of AIS in education. To strengthen the overall concept of preserving natural human presence in the use of AIS, selected ethical and unethical concrete use cases in OA are compared. The use cases are presented in Figure 8, while in the following section they are connected to OA's COU.

### 3.5 Conditions of use

Ethical concerns and resulting “codes of practice” for AIS-assisted educational tools are not entirely new, having been introduced by (Newton and Newton 2019). Nevertheless, OA advances this discourse by reinforcing a human-centric approach through clearly defined COU.

The COU suggest a detailed specification on the visual representation of natural human actors and the intensity of AIS involvement. The underlying ethical considerations and the required low-level properties are addressed in Section 3.4. Based on the characteristic formulations of the “Terms and Conditions of Use” from widely deployed BSD software licenses (de Souza Pinto, 2023), the resulting COU, in accordance with the design summary of OA^4^, are provided in Table 1.

## 4 Tool implementation

**Figure 9:**
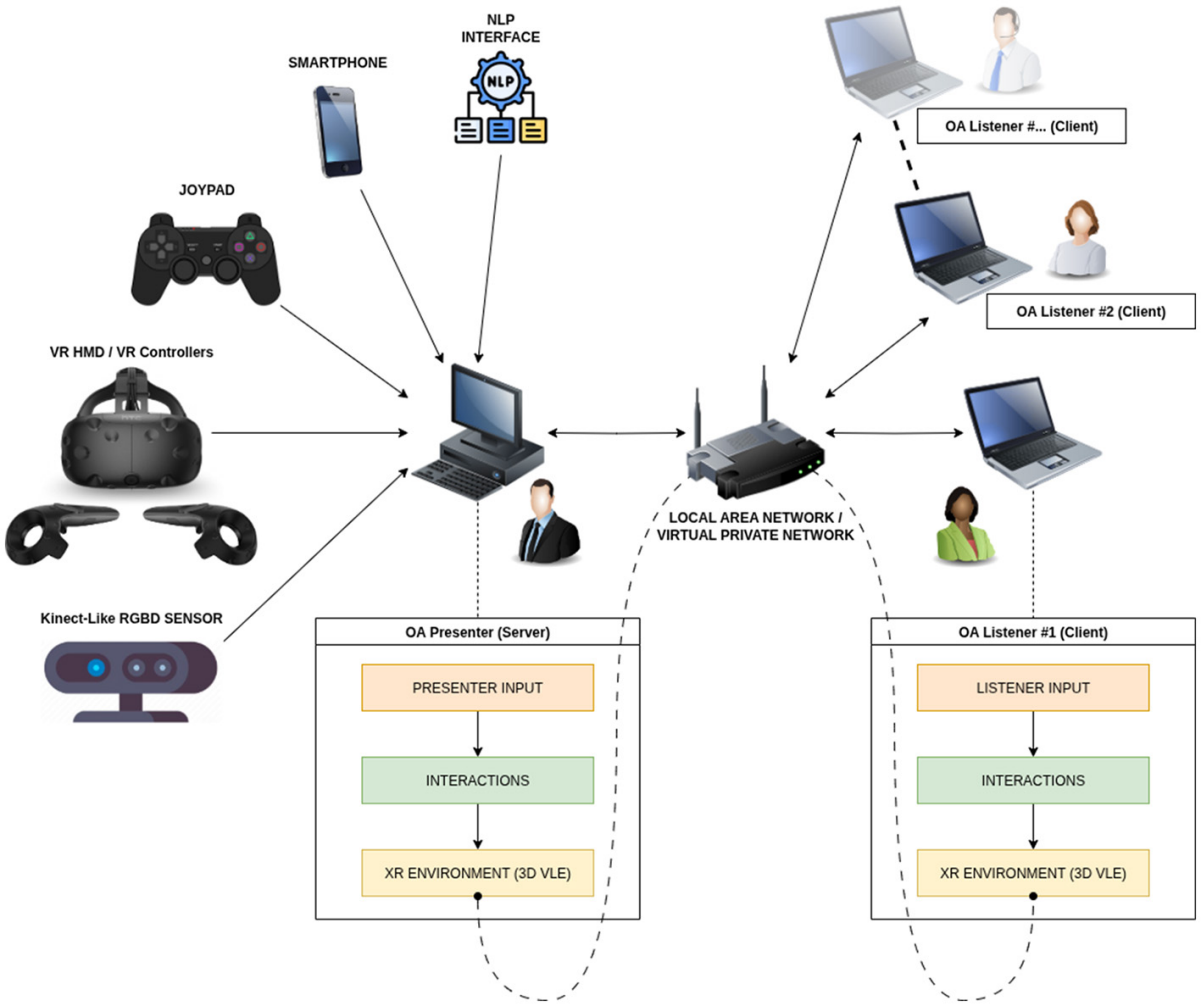
Schematic system overview of OA.^3^

The implementation of OA involves various hardware and software components. The core components are user input devices, commodity computational hardware with a network connection, and various types of displays. A system overview of OA is presented in Figure 9.

### 4.1 Hardware setup

The overall hardware setup of OA depends on the needs of the human actor, and is thus flexible. The recommended setup may utilize commodity hardware (Laptop with an Intel i7 8th generation mobile CPU, four physical cores, 8GB RAM, internal GPU including a network interface, USB interfaces, built-in soundcard and webcam). Additional hardware for advanced user input and visualization may include the following:

- a standard Joypad with 8 axis and various button configurations (e.g, PlayStation controller).
- an Android Smartphone, with the “ROS-Mobile” App running (Rottmann et al., 2020), that may be used to remotely control OA during presenting, or as mobile VR HMD.
- a Kinect-like RGBD Sensor (Rauscher et al., 2015) (e.g., Microsoft Kinect I or ASUS Xtion Pro Live) to enable body/gesture interaction and 3D-visualization of the presenter or audience.
- a VR-HMD, including hand-controllers (e.g., HTC Vive).

**Figure 10:**
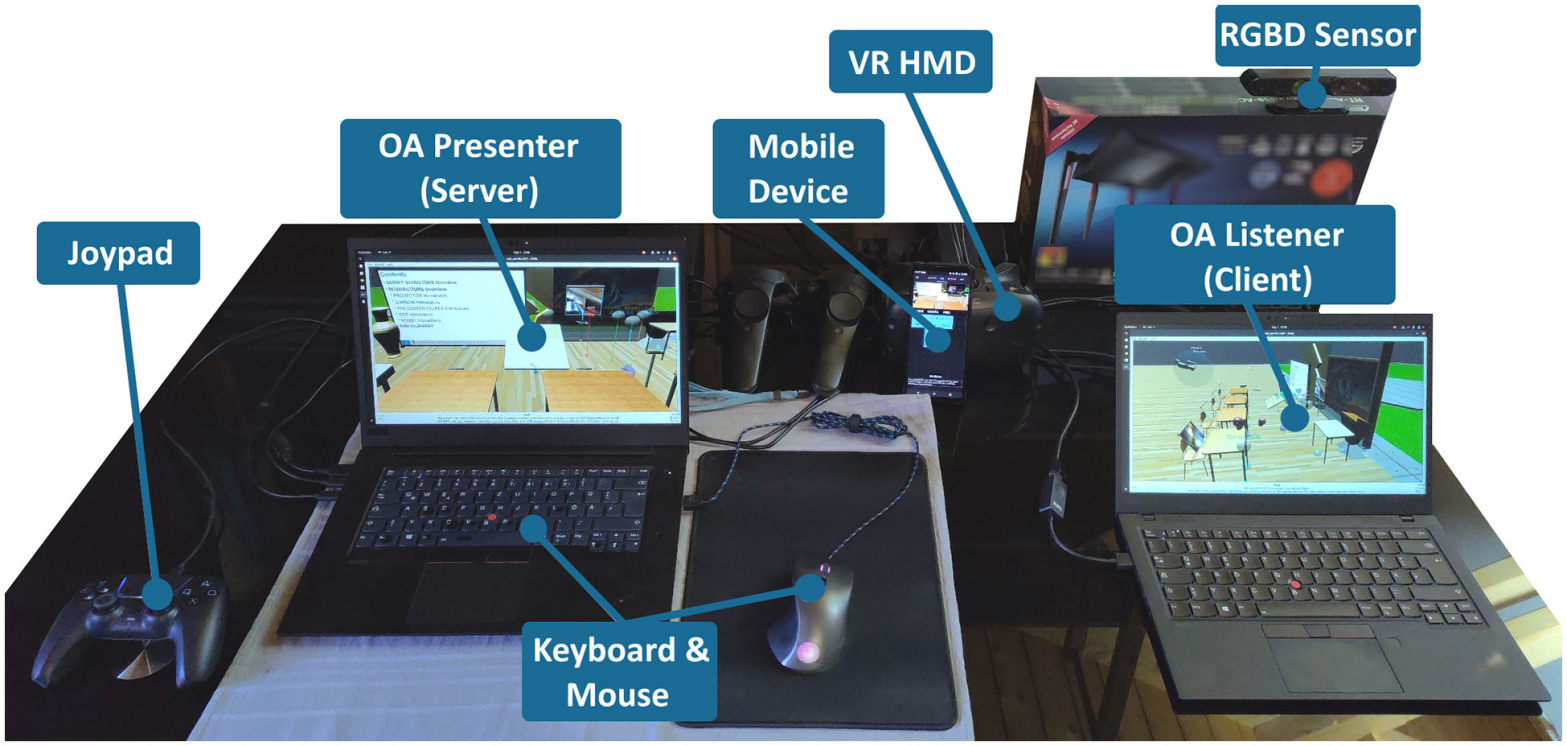
Exemplary hardware setup of OA, including essential input devices and displays for a networked configuration between one OA Presenter and one OA Listener.

An exemplary hardware setup for OA in a networked configuration is shown in Figure 10.

### 4.2 Software architecture

**Figure 11:**
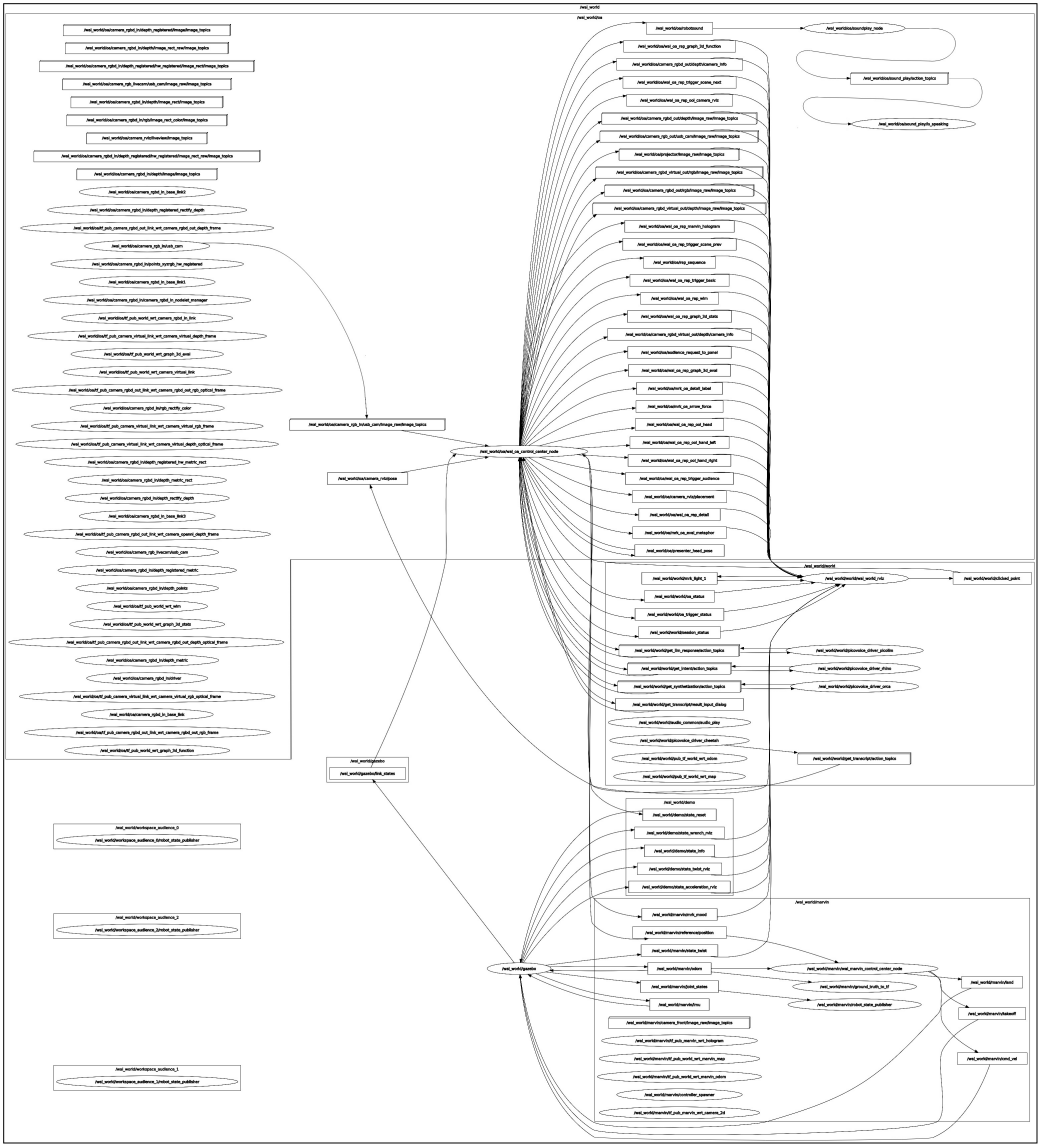
ROS architecture of OA, including essential namespaces, nodes, active topics, and corresponding connections, summarized in a node graph.

Based on the modular architecture of ROS, OA is implemented as ROS node in C++. The node's architecture is built on a singleton pattern (ROS node), whereas user input commands are abstracted (Command Pattern) and forwarded to a state machine (State Pattern). The state machine is combined with a model view controller (MVC) architecture. Additional header-based libraries for visualization in RViz were implemented following an Abstract Factory Pattern. An overview of the ROS architecture of OA, running all relevant ROS nodes for a basic configuration, is shown in Figure 11. OA is currently based on ROS1 Noetic, supported by the Ubuntu 20.04.6 LTS operating system. The source code of OA is publicly available on Github.^5^.

## 5 Conclusions

This article introduces a human-centric, AIS-assisted XR-BL tool, with an open-source software architecture that is publicly available. The tool's ethical COU represent a synthesis between a conceptual low-level framework and established high-level regulations to preserve the natural human presence in the use of AIS. Furthermore, insights into the design process, including the identification of potential ethical implications at use case level, may support the design of future tools.

### 5.1 Discussion and implications

Reflecting on the design and implementation of OA confirmed the extensive effort necessary to identify potential ethical implications of AIS-assisted XR-BL tools on use case level. Thus, it was not possible to cover all use cases and ethical implications in the use of assistive AIS in education. However, essential use cases were identified and substantiated with concrete COU. This could significantly reduce the effort required for the ethical design of related tools in the future. Moreover, the design for different educational levels strongly promoted the concept of an additional property to define the usage intensity of the AIS on a timely basis (IOL), helping decision-makers to facilitate the responsible use of AIS among learners of different ages. Ultimately, by releasing the source code, the intention is to foster further development of human-centric AIS-assisted XR-BL tools as a set of helpful features and plugins are provided back to the open-source community.

### 5.2 Limitations, challenges, and future research

As social interactions between natural human educators and learners mainly occur “in-class” during a teaching/learning session, a corresponding focus was placed on the design summary of OA (see text footnote 4), whereas the framework diagrams (Figures 3–5) model a focus on engineering education at secondary level. Future research and implementations of OA will particularly cover administrative “out-of-class” activities (e.g., automated curricular design), addressing primary and tertiary levels of education. Moreover, despite of that preliminary studies in secondary education indicated a clear positive overall acceptance of the tool during selected teaching/learning sessions (Isop, 2024), the presented work can not provide extensive empirical studies and profound evaluations yet. Consequently, substantial future research includes connecting OA to learning taxonomies and frameworks, such as Bloom's Taxonomy (Forehand, 2010) and the 21st Century Skills Framework (Thornhill-Miller et al., 2023). Complemented by widely accepted scales like the Constructivist Learning Environment Scale (CLES) (Taylor et al., 1995, 1997; Johnson and Mcclure, 2004), or the Community of Inquiry framework (Garrison et al., 2003), OA's pedagogical effectiveness could be assessed throughout measuring indicators like learner engagement, knowledge retention, and critical thinking improvement. To provide empirical evidence, essentially it is planned to conduct classroom observations, further pilot studies, and interviews. Furthermore, since ROS1 officially reached its end of life, a vital immediate step is to migrate to ROS2. Finally, as part of this technology and code article, it was not possible to present use cases for all addressed teaching/learning methods. However, considering richness, diversity, and various connected activities, this could be an exciting challenge for the future of OA.

## Data Availability

The datasets presented in this study can be found in online repositories. The names of the repository/repositories and accession number(s) can be found in the article/supplementary material.

## Footnotes

1 The wallpaper in the background is an adapted image from etsy.com.

2 The symbols of the indication are adapted from “computer” and “AI” images at Flaticon.com.

3 The symbols, representing the user input modalities, are adapted from “nlp interface”, “smartphone”, “joypad”, “virtual reality headset”, and “3d sensor” images at Flaticon.com.

4 The design summary of OA is available online as Google Sheet.

5 The source code of Open Auditorium is publicly available on Github.
